# HLA allele DRB1*01:03: the genetic link to neutralizing IL-10 antibodies in inflammatory bowel disease

**DOI:** 10.1038/s41392-026-03005-8

**Published:** 2026-10-27

**Authors:** Jochen Mattner

**Affiliations:** 1 Mikrobiologisches Institut - Klinische Mikrobiologie, Immunologie und Hygiene, Universitätsklinikum Erlangen and Friedrich-Alexander-Universität (FAU) Erlangen-Nürnberg, Erlangen, Germany; 2 FAU Profilzentrum Immunmedizin (FAU I-MED), FAU Erlangen-Nürnberg, Erlangen, Germany

**Keywords:** Gastroenterology, Diseases

In a recent study published in the *New England Journal of Medicine*, Gharahdaghi and colleagues link the HLA allele DRB1*01:03 to interleukin-10 (IL-10) neutralizing autoantibodies in a cohort of patients with inflammatory bowel disease (IBD).^1^

A complex interplay of environmental factors, intestinal microbiota, and genetic traits underlies IBD, including ulcerative colitis (UC), Crohn´s disease (CD), and IBD that cannot be classified as either UC or CD. Conceptually, it is assumed that these devastating disorders are T cell-driven, with B lymphocytes and (auto-)antibodies playing only a minor role in disease pathogenesis and progression. Moreover, inflammatory cytokine cascades are overrepresented relative to anti-inflammatory cytokine networks, and immune responses to the intestinal microbiota are disrupted.

Genome-wide association studies (GWAS) have identified more than 240 genetic risk loci associated with IBD to date. These include allelic variations affecting various cytokines and cytokine responses. In this context, several loss-of-function variants have been associated with IL-10, a potent anti-inflammatory cytokine and a pivotal mediator of intestinal immune homeostasis.^2^ These include cell-intrinsic variations affecting IL-10 receptor (IL-10R) components or IL-10 signaling pathways (Fig. 1). Monogenic mutations in either one, IL-10R or IL-10, cause very early-onset inflammatory bowel disease. Affected patients develop symptoms during infancy and exhibit a more severe clinical phenotype than polygenic IBD. A few years ago, autoantibodies that neutralize IL-10 were identified in an individual pediatric and one infantile-onset IBD patient who had negative genetic sequencing results for IL-10 or IL-10R defects.^3^ However, a link between these newly identified pathogenic autoantibodies and allelic variations has been lacking.

**Fig. 1:**
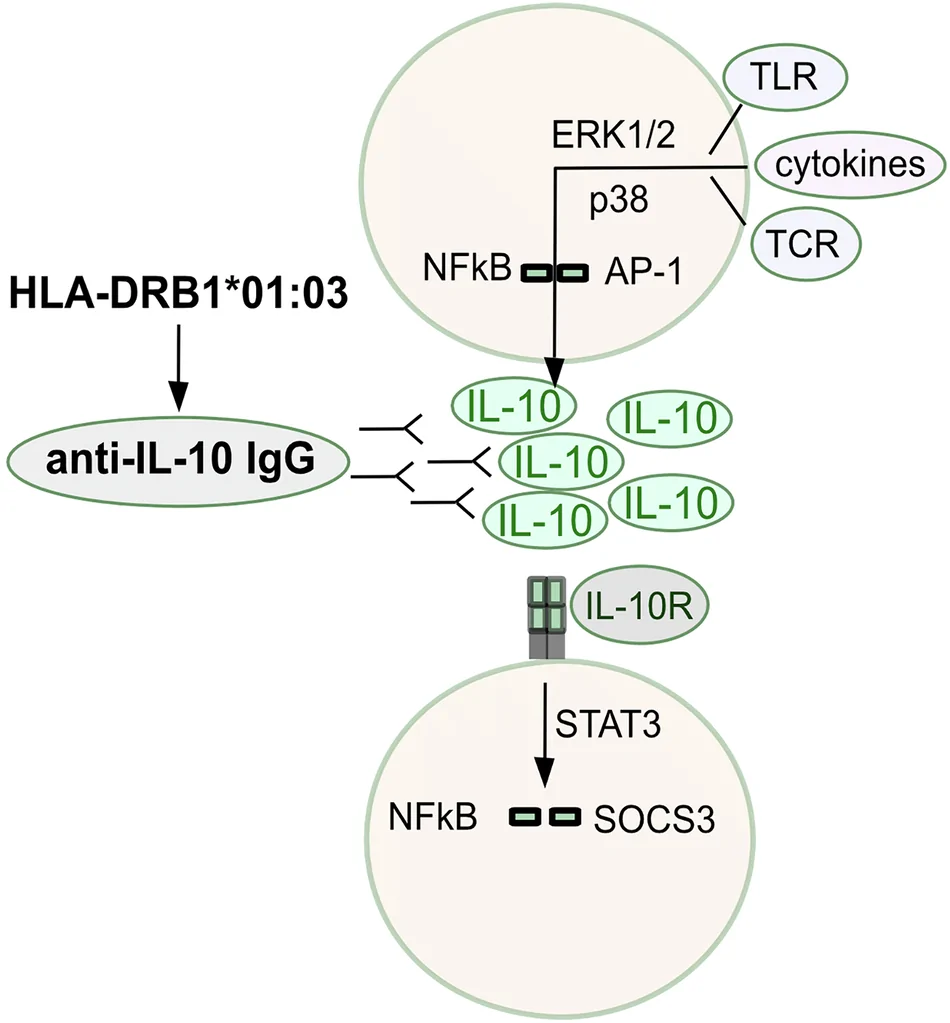
HLA-DRB*01:03 underlies the production of IL-10-neutralizing antibodies. Depending on the cell type, TLR or TCR engagement, as well as various cytokines, can induce IL-10 expression. The current study adds HLA-DRB*01:03 to the pool of genetic variants affecting the action of IL-10 as a potent anti-inflammatory cytokine and gatekeeper of mucosal immune homeostasis. In contrast to the loss of mutant variants described so far that affect IL-10 responsiveness and signal transmission in a cell-intrinsic manner, this genetic link fosters the production of anti-IL10-neutralizing antibodies. Some, but not all, IL-10 Signaling pathways are depicted as examples. α-IL-10 IgG anti-interleukin 10 Immunoglobulin G, AP-1 activator protein 1, Erk1/2 extracellular signal-regulated kinase 1/2, NFkB Nuclear factor kappa B, p38 Mitogen activated protein kinase p38, SOCS3 Suppressor of cytokine signaling 3, STAT3 Signal transducer and activator of transcription 3, TCR T cell receptor, TLR Toll-like receptor

In the current study, these neutralizing IL-10 antibodies have been associated with the HLA-DRB1*01:03 allele,^1^ the strongest risk factor for UC^4^ (Fig. 1). To explore this association, 4909 IBD patients from the Oxford IBD Discovery cohort, the Oxford IBD Validation cohort, and the United Kingdom (U.K.) IBD BioResource cohort have been included. 1006 persons without IBD have been investigated as controls. Serum from these individuals was screened for neutralizing IL-10 IgG antibodies by combining HEK-Blue interleukin-10 reporter assays, which assess downstream cellular responses to interleukin-10, with confirmatory competitive ELISA analyses that measure recombinant interleukin-10. These combined analyses identified 173 patients, or 3.5% of the IBD patients, as positive for IgG antibodies that neutralize IL-10. Accordingly, 5.3% in the Oxford IBD Discovery cohort, 3.1% in the Oxford IBD Validation cohort, and 3.2% in the U.K. IBD BioResource cohort were anti-IL-10 IgG-positive. Neutralizing anti–interleukin-10 autoantibodies were detected in 2.5% of CD patients, in 4.4% of UC patients, and in 5.9% of unclassified IBD patients. Follow-up studies ranging from 0.3 to 14 years (8.6 ± 4.5 years) showed long-term persistence of the neutralizing anti–interleukin-10 IgG levels in 7 of 10 patients (70%). Unfortunately, no information about disease progression/remission or responsiveness to commonly used treatment protocols is provided in patients who have become autoantibody negative. In contrast to the IBD patients, none of the controls showed anti-IL-10 autoantibodies, based on the combined results of the cellular IL-10 neutralization assay and the confirmatory competitive ELISA.

The autoantibodies against IL-10 were specific. One patient had anti-IL-6 antibodies, and two patients had anti-tumor necrosis factor (TNF) antibodies, as detected by the Randox Chemiluminescence assay following previous treatment with infliximab or adalimumab. In the Luminex Fluorescence assay, two individuals in the IBD cohort showed autoantibodies against IFN-γ.

Cytokine concentrations are usually low in the sera of IBD patients even during acute disease flares. Therefore, peripheral blood mononuclear cells (PBMCs) from healthy controls were exposed to sera from IBD patients and stimulated with lipopolysaccharide (LPS). Here, anti-IL-10 activity was associated with increased release of inflammatory cytokines, including IL-23, IL-1β, TNF, and IL-6, reflecting the aberrant and overzealous immune responses to bacterial antigens characteristic of IBD. No further controls, including autologous PBMC-sera pairs, were included.

To assess a potential link between anti-IL-10 antibodies and the HLA genotype, a multiancestry HLA imputation was applied to 404 patients of European ancestry from the Oxford cohort. Accordingly, the strongest allele associated with anti–interleukin-10 antibodies was identified in the HLA-DRB1 locus as HLA-DRB1*01:03. This association between anti-IL-10 IgG responses and HLA-DRB1*01:03 was validated using multiancestry HLA imputation genotyping data from the U.K. IBD BioResource cohort. Using all available haplotype data, 13.6% of patients in the Oxford cohort and 3.9% of the English population were identified as positive for the HLA-DRB1*01:03 allele.

In summary, HLA-DRB1*01:03, which is strongly linked to UC, could be associated with the production of IL-10-neutralizing IgG autoantibodies. Environmental factors, gastrointestinal infections, IBD flares, or infections that trigger autoimmunity, potentially through molecular mimicry, may drive the loss of tolerance to interleukin-10. It remains unclear, however, why some carriers of this HLA variant develop autoantibodies while others don´t. In addition, there is no explanation for why screening was narrowed to the reporter assays and the ELISA, even though particle-based flow cytometry assays, another cellular IL-10 reporter assay, and a cytokine release assay were initially included in the analyses.

The identification of neutralizing interleukin-10 IgG autoantibodies in a subgroup of IBD patients and their strong association with HLA-DRB1*01:03 may open up new diagnostic, prognostic, and therapeutic opportunities. Thus, screening for HLA-DRB1*01:03 and autoantibodies may avoid ineffective management of IBD with standard anti-inflammatory and/or immunosuppressive medications. In this context, several therapeutic options targeting B lymphocytes are available. For example, different antibodies, such as anti-CD20 therapy, have been successfully applied in one of the initially identified infantile-onset IBD patients with anti-IL10 neutralizing antibodies,^3^ or chimeric antigen receptor (CAR) T cell therapy has depleted B cells in a patient with massive B cell infiltration and promoted clinical remission.^5^ As both patients were refractory to standard treatment protocols, these data indicate that this subgroup of IBD patients requires therapies that target B-cell-mediated autoantibody production specifically or specific alternative pathways that detour IL-10. Thus, understanding pathogenic pathways shared by rare genetic defects, and identifying disrupted cellular and molecular circuits in acquired autoimmunity and immune-mediated diseases, is pivotal for personalized therapeutic intervention.

## References

[1] Gharahdaghi, N. et al. Interleukin-10 Autoantibodies and HLA-DRB1*01:03 in inflammatory bowel disease. *N. Engl. J. Med.* **394**, 2212–2222 (2026). 10.1056/NEJMoa2513654 42269151

[2] Xiao, J., Ye, Z. & Huang, Y. A Comprehensive Review of IL-10 and IL-10 Receptor Deficiencies: From Basic Science to Clinical Bedside. *Clin. Rev. Allergy Immunol.* **69**, 25 (2026). 10.1007/s12016-026-09154-6 PMC13021692 41888292

[3] Griffin, H. et al. Neutralizing autoantibodies against Interleukin-10 in Inflammatory Bowel Disease. *N. Engl. J. Med.* **391**, 434–441 (2024). 10.1056/NEJMoa2312302 PMC7616361 39083772

[4] Vestergaard, M. V. et al. HLA-DRB1*01:03 and Severe Ulcerative Colitis. *JAMA* **332**, 1941–1943 (2024). 10.1001/jama.2024.20429 PMC11480900 39403737

[5] Muller, F. et al. CD19 CAR T-Cell therapy in multidrug-resistant ulcerative colitis. *N. Engl. J. Med.* **393**, 1239–1241 (2025). 10.1056/NEJMc2508023 40991907

